# Supplementary material for: Free charge photogeneration in a single component high photovoltaic efficiency organic semiconductor
Source: Nat Commun. 2022 May 20;13:2827. doi: 10.1038/s41467-022-30127-8 (PMC9122989; doi:10.1038/s41467-022-30127-8)
Supplement: Supplementary file 1 — Supplementary Information [file 41467_2022_30127_MOESM1_ESM.pdf]

Supplementary Information: Free Charge Photogeneration in a Single Component High Photovoltaic Efficiency Organic Semiconductor

Michael B. Price[a,b,1,*], Paul A. Hume[a,b,1,*], Aleksandra Ilina[a,b], Isabella Wagner[a,b], Ronnie R. Tamming[a,b], Karen E. Thorn[a,b], Wanting Jiao[c], Alison Goldingay[d], Patrick J. Conaghan[d], Girish Lakhwani[d], Nathaniel J.L.K. Davis[a,b], Yifan Wang[e,f], Peiyao Xue[e], Heng Lu[e], Kai Chen[a,b], Xiaowei Zhan[e], Justin M. Hodgkiss[a,b,*]

[a] School of Chemical and Physical Sciences, Victoria University of Wellington, Wellington, New Zealand

[b] MacDiarmid Institute for Advanced Materials and Nanotechnology, New Zealand.

[c] Ferrier Research Institute, Victoria University of Wellington, Wellington, New Zealand

[d]ARC Centre of Excellence in Exciton Science, School of Chemistry, University of Sydney, NSW 2006, Australia

[e] School of Materials Science and Engineering, Peking University, Beijing 100871, China.

[f] College of Materials Science and Engineering, Qingdao University, Qingdao 266071, China

* Correspondence should be sent to J.M.H. (Justin.Hodgkiss@vuw.ac.nz), P.A.H. (Paul.Hume@vuw.ac.nz), and M.B.P. (Michael.price@vuw.ac.nz)

[1] Authors contributed equally to this work

Table of contents

Supplementary Figure 1. Chemical structures, names, and abbreviations for all materials discussed in the main manuscript. 4

Supplementary Figure 2. TA spectra and kinetics of Y6 in solid solution of polystyrene versus neat film. 5

Supplementary Figure 3. UV-vis spectra of Lewis acid-doped films of Y6 showing polaron signature and visible range transient absorption spectra of Y6 films. 5

Supplementary Figure 4. Fluence dependent exciton kinetics from transient absorption. 6

Supplementary Figure 5. Excitation energy-dependent exciton kinetics from transient absorption. 6

Supplementary Figure 6. Evidence of long-lived triplets in Y6 neat thin film transient absorption. 7

Supplementary Figure 7. Current density-voltage (J-V) curves for single component Y6 devices with different hole extraction layers. 7

Supplementary Figure 8. Intensity dependence of short-circuit current density in a low range – from 0.024 to 0.1 suns. 8

Supplementary Figure 9. J-V curves for Y6 devices with very low donor content of PTB7-Th. 8

Supplementary Figure 10. Exciton, electron, and hole dynamics in a 1:1.2 PTB7-Th:Y6 blend measured by transient absorption. 9

Supplementary Figure 11. Exciton and charge kinetics for neat Y6 compared to Y6 blended with 0.2 weight fraction PCBM. 9

Supplementary Figure 12. Molecular geometries for electronic coupling calculations. 10

Supplementary Figure 13. Ionization energy (IE) and electron affinity (EA) calculated using long-range polarizable embedding of charges in a model thin film. 11

Supplementary Table 1. Energy levels and electronic couplings for localized exciton and CT states. 12

Supplementary Note 1. Intensity-dependent photoluminescence measurements on Y6 thin films. 13

Supplementary Note 2. Consideration of exciton trap filling as an explanation of increasing photoluminescence efficiency with increasing fluence. 14

Supplementary Figure 14. Comparison of intensity-dependent photoluminescence to state-filling model, varying trap concentration Q_0. 15

Supplementary Figure 15. Comparison of intensity-dependent photoluminescence to state-filling model. 15

Supplementary Figure 16. Comparison of predicted exciton decay kinetics from trap state-filling model, with transient absorption data at different excitation densities. 17

Supplementary Note 3. Comment on triplet yields in neat Y6 19

Supplementary Note 4. Optical-pump-terahertz-probe spectroscopy 20

Supplementary Figure 17. Optically-pumped time-resolved terahertz absorption kinetic of neat Y6, compared to transient absorption kinetic decays of excitons and charges. 21

Supplementary Note 5. Kinetic models of exciton and free charge signals in transient absorption and fluence dependent PLQE 22

Basic Model 22

Supplementary Figure 18. Exciton dissociation, and steady state charge fractions with error of fit to TA and PL data for the Basic Model. 24

Model Including Triplet-Triplet Annihilation 25

Supplementary Figure 19. Kinetic model, including triplet-triplet annihilation, of charge generation in Y6 with transient absorption, PLQE and predicted steady-state free charge fractions. 26

Supplementary Figure 20. Exciton dissociation, and steady state charge fractions with error of fit to TA and PL data for the Basic Model with TTA included. 27

Model Including Finite Charge Trap Population 28

Supplementary Figure 21. Kinetic model, including hole trap saturation, of charge generation in Y6 with transient absorption, PLQE and predicted steady-state free charge fractions. 29

Supplementary Figure 22. Exciton dissociation, and steady state charge fractions with error of fit to TA and PL data for the Basic Model with hole traps included. 30

Consideration of Explicit Singlet-CT State Equilibrium 30

Supplementary Note 6. Photon Reabsorption in thin films 31

Supplementary Figure 23. Simulated PLQE of Y6 with and without the effect of photon recycling. 31

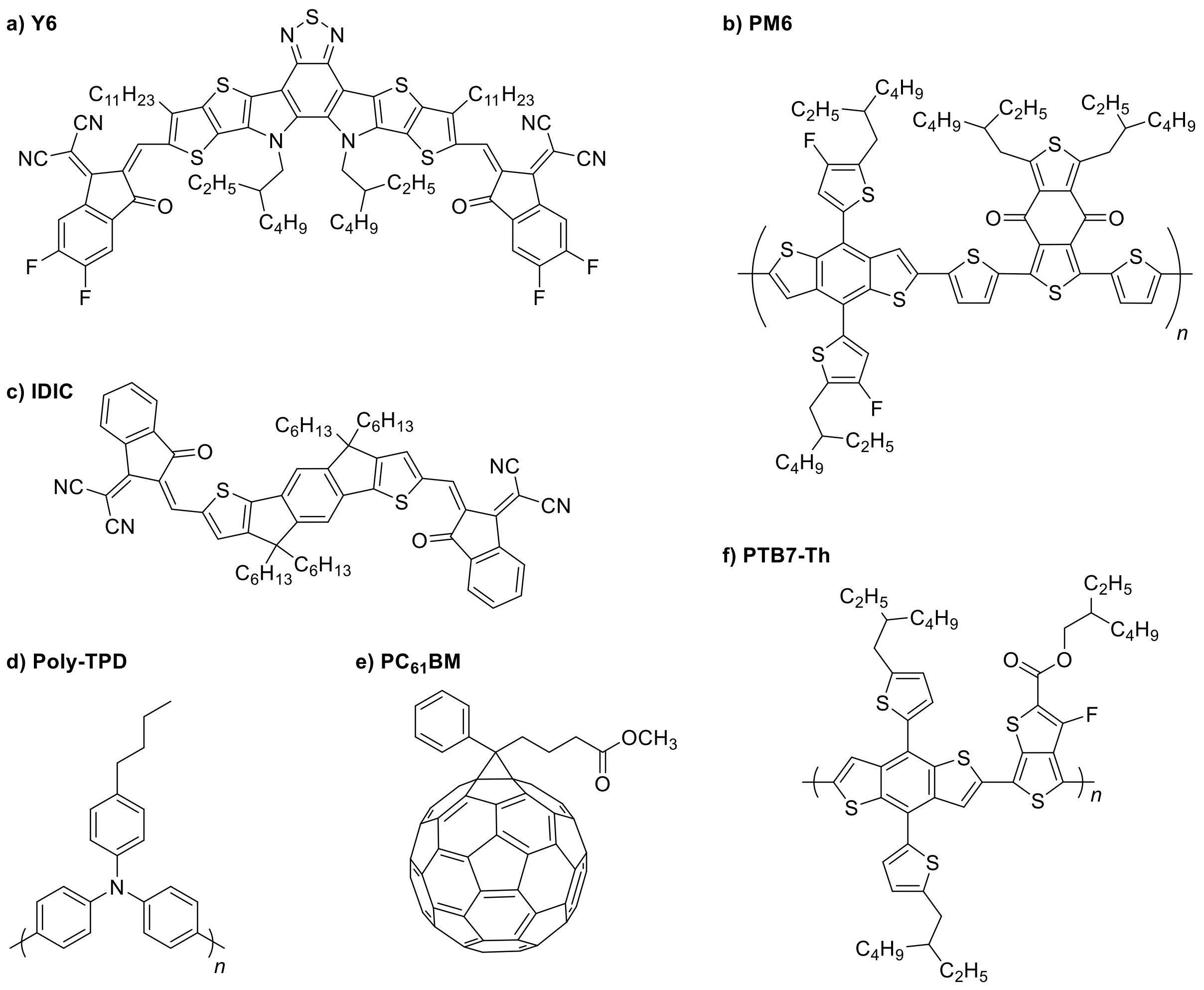

Supplementary Figure 1. Chemical structures, names, and abbreviations for all materials discussed in the main manuscript. A) 2,20-((2Z,20Z)-((12,13-bis(2-ethylhexyl)-3,9-diundecyl-12,13-dihydro-[1,2,5]thiadiazolo[3,4-e]thieno[2,"30':4',50]thieno[20,30:4,5]pyrrolo[3,2-g]thieno[20,30:4,5]thieno-[3,2-b]indole-2,10-diyl)bis(me-thanylylidene))bis(5,6-difluoro-3-oxo-2,3-dihydro-1H-indene-2,1-diylidene))dimalononitrile (**Y6**)[1], **b)** Poly[(2,6-(4,8-bis(5-(2-ethylhexyl-3-fluoro)thiophen-2-yl)-benzo[1,2-b:4,5-b']dithiophene))-alt-(5,5-(1',3'-di-2-thienyl-5',7'-bis(2-ethylhexyl)benzo[1',2'-c:4',5'-c']dithiophene-4,8-dione)) (**PM6**)[2],
c) 2,2'-[(4,4,9,9-Tetrahexyl-4,9-dihydro-s-indaceno[1,2-b:5,6-b']-dithiophene-2,7-diyl)bis[methylidyne(3-oxo-1H-indene-2,1(3H)-diylidene)]]bis-propanedinitrile (**IDIC**)[3],
d) Poly(4-butyltriphenylamine) (**Poly-TPD**), **e)** Phenyl-C61-butyric acid methyl ester (**$PC_{61}BM$**), **f)** Poly[4,8-bis(5-(2-ethylhexyl)thiophen-2-yl)benzo[1,2-b;4,5-b']dithiophene-2,6-diyl-alt-(4-(2-ethylhexyl)-3-fluorothieno[3,4-b]thiophene-)-2-carboxylate-2-6-diyl]] (**PTB7-Th**)[4].

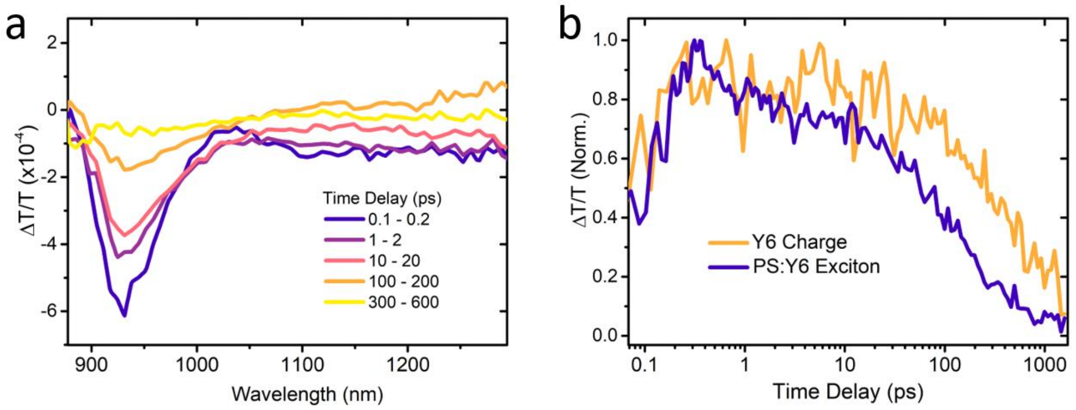

Supplementary Figure 2. TA spectra and kinetics of Y6 in solid solution of polystyrene versus neat film. a) Time-slices of Y6 in a solid solution of polystyrene (1:50 weight ratio), at low excitation fluence of ~0.5 µJcm-2. The spectra is markedly different to the thin-film spectra shown in main text Figure 2a, where there is no longer-lasting red-shifted negative peak, indicating only excitons are present. B) Kinetics of the excitonic peak of the Y6 in polystyrene, in blue, versus the extracted charge/CT kinetics from a neat thin film of Y6 showing a much longer decay.

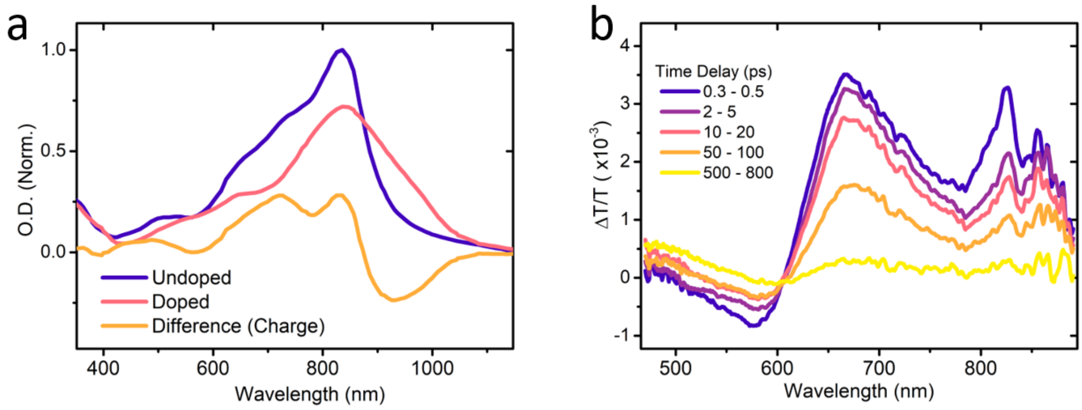

Supplementary Figure 3. UV-vis spectra of Lewis acid-doped films of Y6 showing polaron signature and visible range transient absorption spectra of Y6 films. a) UV-vis spectra of undoped Y6 (blue line) and doped Y6 film (pink line) from Wang *et al.*[11] normalised to the red tail at 1100 nm. The orange line shows the difference between the two spectra, which gives another representation of charge (polaron) spectra, which matches well with the proposed charge spectra ascertained from transient absorption measurements. **b)** Visible transient absorption spectra of a neat film of Y6, pumped with 550 nm, 200 fs, excitation, at ~5×10^{17} excitations/cm^3. The spectral region beyond 800 nm is significantly more noisy than other spectral regions due to the presence of the fundamental 800 nm pump, at decreased whitelight probe stability. However the spectral shape matches well with the expected polaron visible features from steady-state spectroscopy shown in Supplementary Fig. 3a.

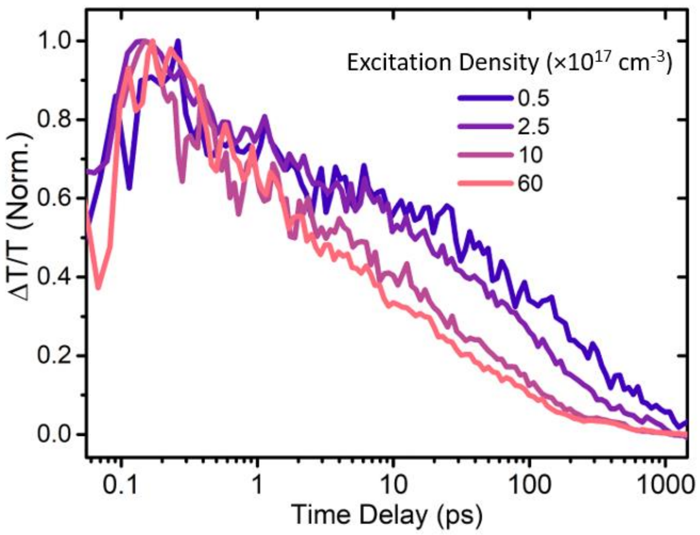

Supplementary Figure 4. Fluence dependent exciton kinetics from transient absorption. A fast fluence independent decay is present in the first picosecond of the exciton kinetics, extracted by genetic algorithm from transient absorption measurements at specified excitation intensities, pumped with 700 nm pulses.

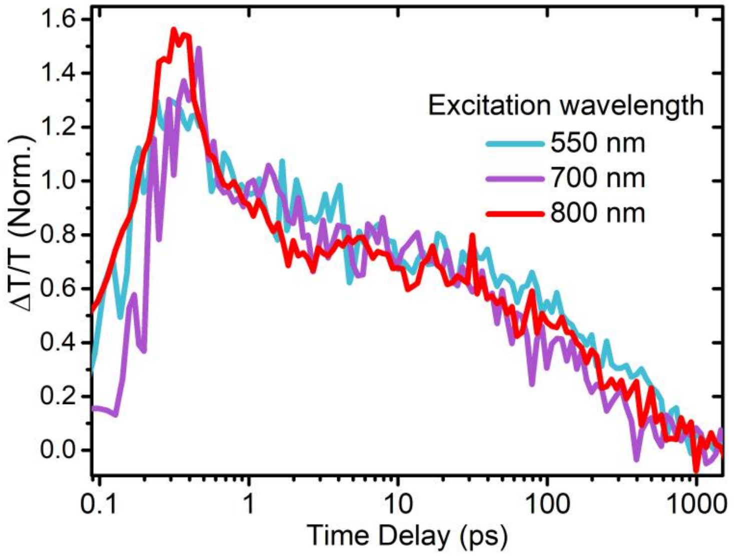

Supplementary Figure 5. Excitation energy-dependent exciton kinetics from transient absorption. Pumped with intensities giving approximately 2.5×10^{17} carriers/cm^3, the exciton kinetics are the same for the different excitation wavelengths.

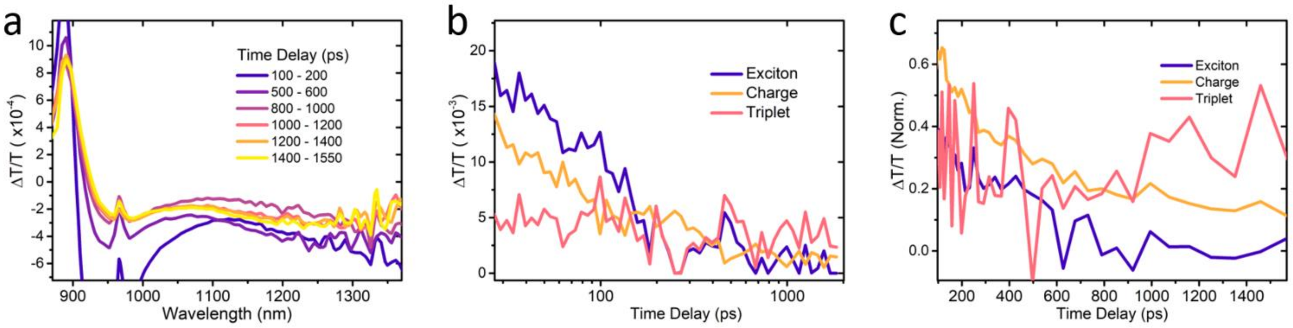

Supplementary Figure 6. Evidence of long-lived triplets in Y6 neat thin film transient absorption. a) Time-slices of un-normalised spectra at later times (beyond 100 ps) of neat Y6 excited at 5×10^{17} carriers/cm^3, with 700 nm, 150 fs pulses. A rise in the broad negative signal, consistent with the triplet signature identified by Gillet *et al*.[12] around 1200 nm, can be seen after ~800 ps. This feature at 1200-1250 nm is the blue edge of the 1450 nm PIA peak identified by Gillet *et al.* but low signal beyond 1250 nm obscures our data at higher wavelengths. **b)** Exciton, charge and triplet kinetics extracted from use of a genetic algorithm to the spectra in Supplementary Fig. 6a. The triplet kinetic is dominant after ~500 ps, though the signal is noisy. **c)** Exciton, charge and triplet kinetics taken from the peak positions of the respective species (915 nm, 980 nm, and 1200 nm), normalised, showing the growth of the triplet species after 600 ps while the charge species decays.

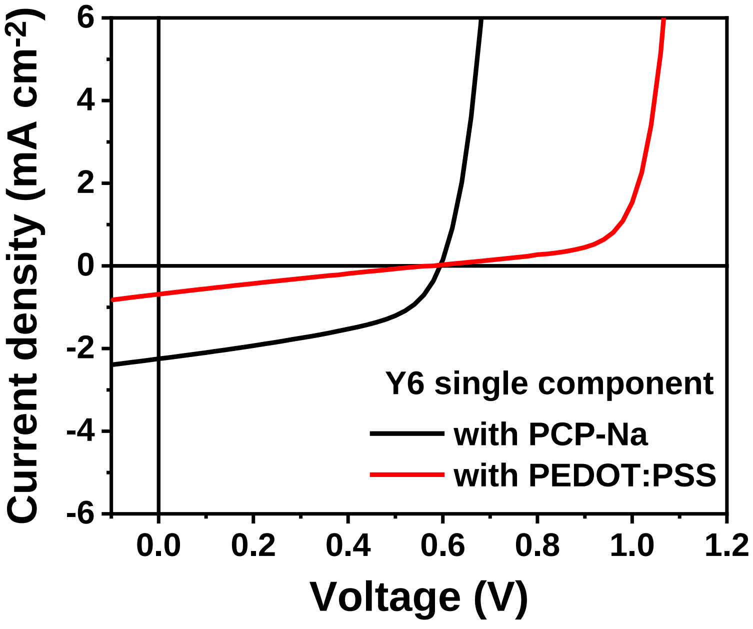

Supplementary Figure 7. Current density-voltage (J-V) curves for single component Y6 devices with different hole extraction layers. With PEDOT:PSS, PCE of the best performing device pixel was 0.09%. With PCP-Na,[23] the best performing device achieved 0.63% PCE.

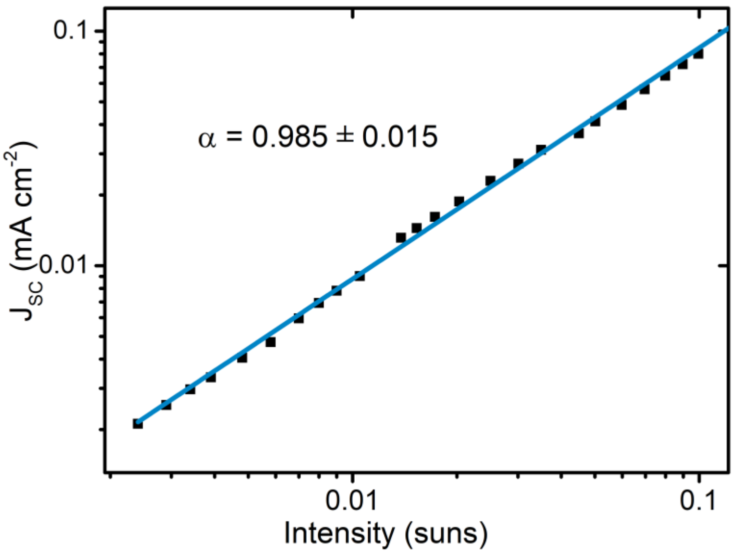

Supplementary Figure 8. Intensity dependence of short-circuit current density in a low range – from 0.024 to 0.1 suns. The blue line shows a fit to the current vs intensity with an exponent of 0.985, indicating small bimolecular and space-charge recombination losses at these very low fluences.

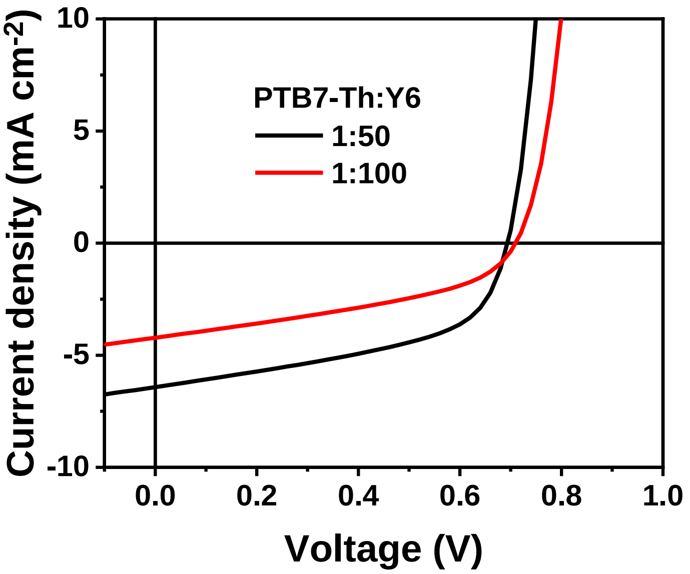

Supplementary Figure 9. J-V curves for Y6 devices with very low donor content of PTB7-Th. For the device with a ratio of PTB7-Th:Y6 ratio of 1:100, power conversion efficiency was 1.23%, and for the 1:50 blend, PCE was 2.26%.

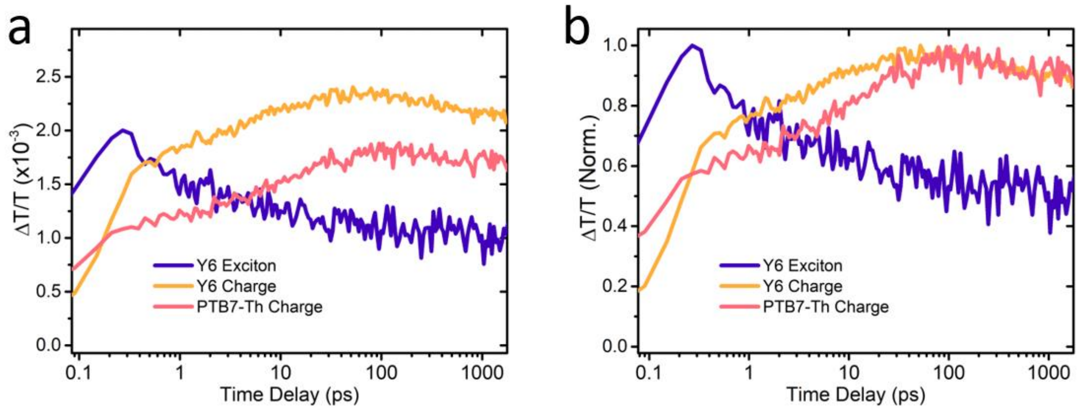

Supplementary Figure 10. Exciton, electron, and hole dynamics in a 1:1.2 PTB7-Th:Y6 blend measured by transient absorption. a) un-normalised transient absorption measurements of a blend film excited with 800 nm, 150 fs pulses, at $1x10^{17}$ carriers/cm^3. Exciton, Y6 charge, and PTB7-Th charge kinetics are attained by using the genetic algorithm described in the main text, with mask spectra also described in main text Fig 2. As expected, the PTB7-Th hole lags the Y6 charge signature rise. And the Y6 charge rise shows a higher prompt rise than the PTB7-Th charge prompt rise **b)** Normalised kinetics of those shown in Supplementary Fig. 10a.

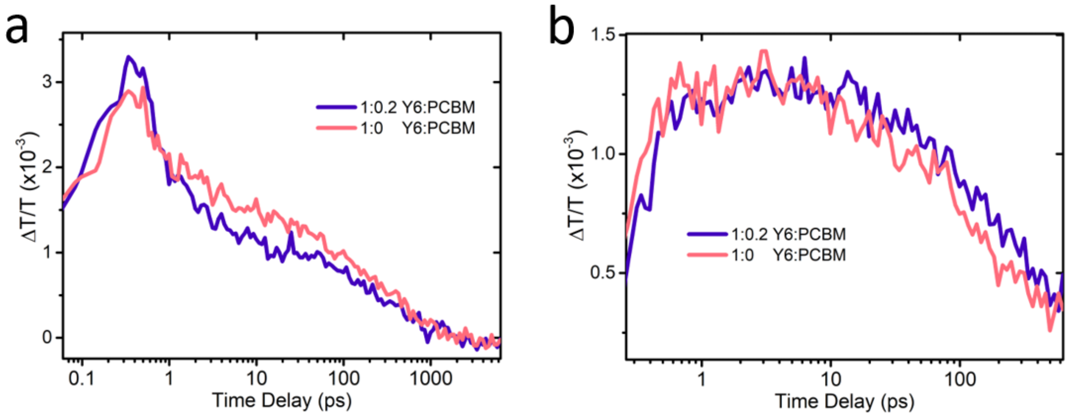

Supplementary Figure 11. Exciton and charge kinetics for neat Y6 compared to Y6 blended with 0.2 weight fraction PCBM. a) Un-normalised exciton kinetics from transient absorption of neat Y6 and Y6:PCBM measured at the same excitation density, approx. $5x10^{17}$ cm^{-3}, excited with 800 nm, 150 fs pulses. **b)** the corresponding charge kinetics. While the exciton decay is accelerated for the Y6 PCBM blend between 1- 800 ps compared to the neat Y6, the charge decay is slowed in the blend film compared to the neat film. This supports the hypothesis that increased quadrupolar fields in the blend film can delay charge recombination, and shift the dynamic exciton-charge equilibrium slightly towards charges.

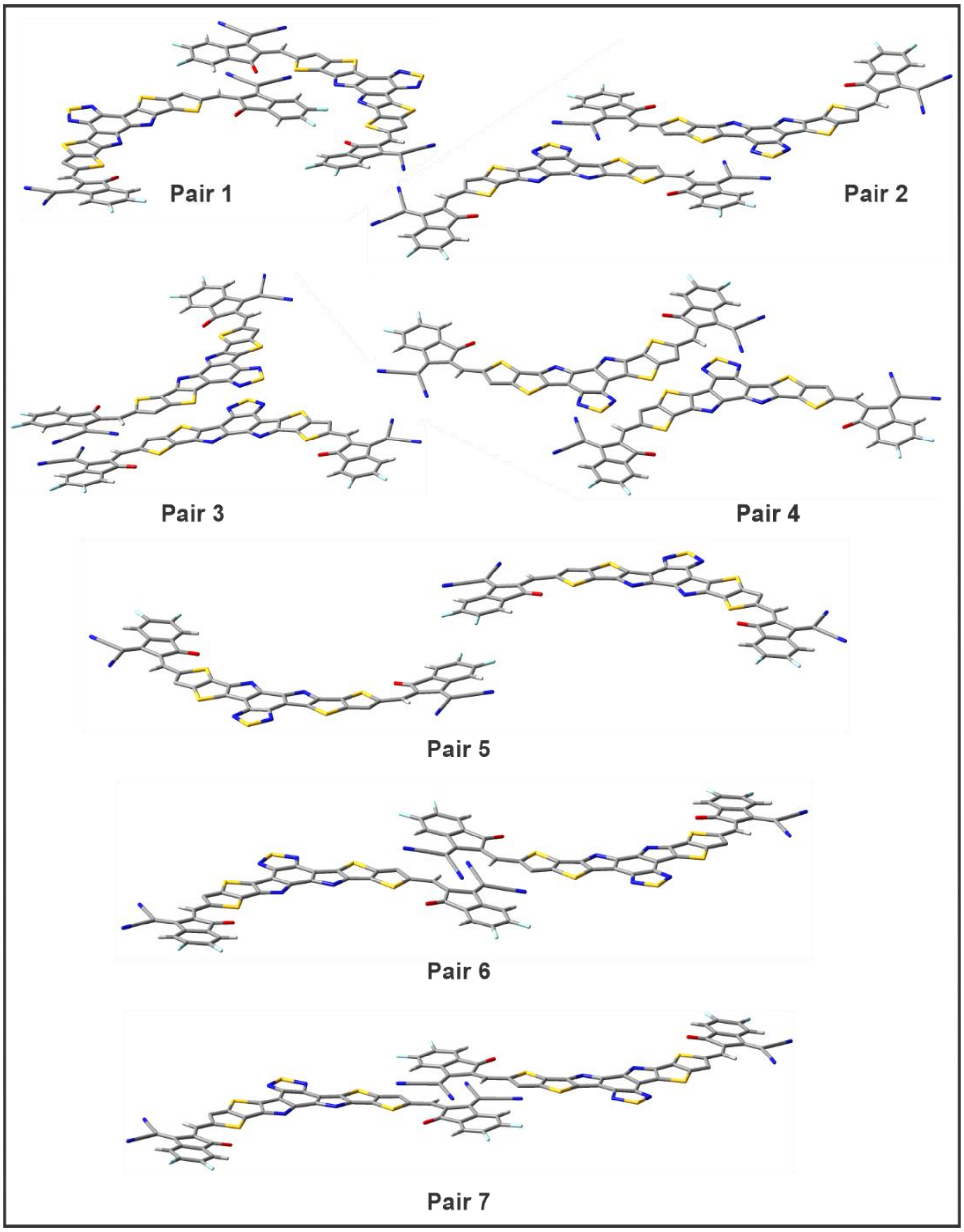

Supplementary Figure 12. Molecular geometries for electronic coupling calculations. Calculations were performed on π-stacked molecular pairs extracted from the Y6 crystal structures, with alkyl chains truncated to methyl to reduce computational expense (omitted for clarity in this Figure).

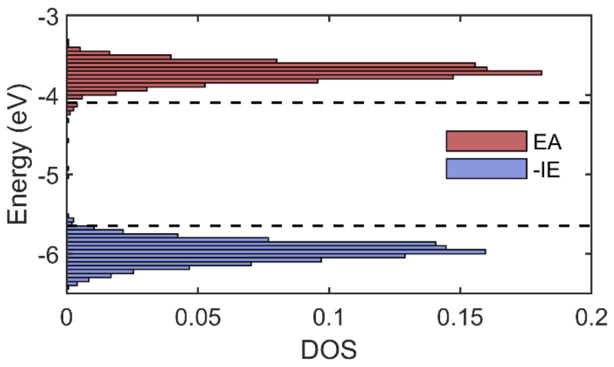

Supplementary Figure 13. Ionization energy (IE) and electron affinity (EA) calculated using long-range polarizable embedding of charges in a model thin film. Calculations were performed for all molecules (1536 in total) in a 10 nm thick model thin film based on molecular dynamics equilibration of the Y6 crystal structure. Dashed lines indicate the DOS onsets determined by photoelectron spectroscopy in air (-IE), and inverse photoemission spectroscopy (EA).[26]

Pair 1	E (eV)	|V| (meV)				
		Ex₁	Ex₂	CT₁	CT₂	GS
**Ex_1**	1.73	-	48	37	80	9
**Ex_2**	2.01	48	-	90	33	6
**CT_1**	1.69	37	90	-	6	77
**CT_2**	2.01	80	33	6	-	49

Pair 2	E (eV)	|V| (meV)				
		Ex_1	Ex_2	CT_1	CT_2	GS
**Ex_1**	1.71	-	29	36	5	7
**Ex_2**	1.72	29	-	4	35	8
**CT_1**	1.53	36	4	-	10	83
**CT_2**	1.54	5	35	10	-	83

Pair 3	E (eV)	|V| (meV)				
		Ex_1	Ex_2	CT_1	CT_2	GS
**Ex_1**	1.74	-	33	75	71	10
**Ex_2**	2.03	33	-	64	48	9
**CT_1**	1.71	75	64	-	3	73
**CT_2**	1.92	71	48	3	-	23

Pair 4	E (eV)	|V| (meV)				
		Ex_1	Ex_2	CT_1	CT_2	GS
**Ex_1**	2.04	-	14	10	46	9
**Ex_2**	2.04	14	-	46	10	8
**CT_1**	1.96	10	46	-	3	67
**CT_2**	1.96	46	10	3	-	67

Pair 5	E (eV)	|V| (meV)				
		Ex_1	Ex_2	CT_1	CT_2	GS
**Ex_1**	2.02	-	16	3	13	2
**Ex_2**	2.02	16	-	13	3	2
**CT_1**	2.14	3	13	-	0	5
**CT_2**	2.14	13	3	0	-	5

Pair 6	E (eV)	|V| (meV)				
		Ex_1	Ex_2	CT_1	CT_2	GS
**Ex_1**	1.74	-	36	29	60	7
**Ex_2**	1.74	36	-	58	30	6
**CT_1**	1.76	29	58	-	5	58
**CT_2**	1.76	60	30	5	-	63

Pair 7	E (eV)	|V| (meV)				
		Ex_1	Ex_2	CT_1	CT_2	GS
**Ex_1**	1.99	-	38	35	26	8
**Ex_2**	2.00	38	-	26	35	8
**CT_1**	2.02	35	26	-	1	33
**CT_2**	2.02	26	35	1	-	32

Supplementary Table 1. Energy levels and electronic couplings for localized exciton and CT states. Localized/diabatic states were obtained from a separate calculation in which the molecules were separated from one another by 10 Å. At this distance, interactions involving orbital overlap are negligible, resulting in the formation of localized CT states. Excitonic states were obtained by applying the fragment excitation difference diabatization scheme to the adiabatic states of the reference system.[24] The non-orthogonality of the localized states in the crystal geometry is accounted for by application of a Löwdin orthogonalization, however we note that the effect of this procedure on the calculated values is minor in the present case.[25]

Supplementary Note 1. Intensity-dependent photoluminescence measurements on Y6 thin films.

Figure 1 a in the main text shows the change in PL efficiency as a function of excitation density of Y6 thin films. Due to the higher error associated with standard PLQE measurements performed in an integrating sphere (mainly due to inherent laser instability), we use a different method as a proxy to gain a more accurate gauge of fluence dependent PL efficiency changes. We measure the PL in a standard PL configuration, using lenses to focus into an InGaAs detector to maximise captured photoluminescence counts. We use 2 longpass 650 nm filters before the entrance slit to the detector to minimise excess pump scatter. To account for inherent laser power variation, we use the ratio of the peak of the second harmonic pump scatter signal divided by the integrated PL signal. The error associated with this, as shown in Figure 1 a, is given by:

$$\sigma = \sqrt{\sum_i \left(\left| S_{norm_i} \right| \sqrt{\frac{{\sigma_S}^2 + {\sigma_B}^2}{{S_i}^2} + \frac{{\sigma_S}^2 + {\sigma_B}^2}{P^2}} \right)^2} \qquad (S1)$$

Where S_i is the raw PL counts for every pixel/wavelength, i. σ_S and σ_B are the standard deviations of the signal and background (shot noise), P is the peak scatter counts of the second harmonic, and S_{norm} is the signal divided by the peak scatter. This represents the shot-to-shot error in the normalised PLQE.

In Figure 3 c, to estimate the absolute, rather than relative, PLQE, we have multiplied the normalised values from Figure 1 a by the average peak value obtained from measurements done by the standard integrating sphere technique of de Mello *et al*.[5] From our standard PL measurements, we measure that PL intensity drops by a factor of ~2 when measured in air rather than vacuum, hence we have multiplied our values by this factor as well, as the integrating sphere measurements were performed in air. There is hence significantly more error associated with those PLQE values presented in Figure 3 c, arising from variation due to laser power fluctuation, environmental effects on thin films, measurements of film thickness with the DekTak profilometer, and estimates of photon reabsorption.

Supplementary Note 2. Consideration of exciton trap filling as an explanation of increasing photoluminescence efficiency with increasing fluence.

Excitonic Trap State-Filling Model

One potential explanation for the intensity-dependent PL is trap state-filling. In this scenario a population of excitonic trap sites quenches a significant fraction of photo-excitations at low excitation densities. As the intensity increases, trap sites are filled faster than they can be regenerated, leading to an increase in the relative PL intensity. Beyond a certain excitation density, the traps become saturated and the PL yield ceases to rise. When singlet-singlet annihilation at high fluences is also considered, an initial rise, followed by a decrease, can be obtained. In this section, we demonstrate that large quenching rates are required for our data to fit this model, which conflicts with the transient absorption (TA) data, and with the reported exciton diffusion coefficient for Y6, $D = 0.054\ \mathrm{cm^2s^{-1}}$ (which should be valid if charges are not formed in Y6.[6])

The rate equations for this scenario are:

$$\frac{dS}{dt} = I_0 - \left(\frac{1}{\tau} + k_{quench}Q\right)S - k_b S^2 \qquad (S2)$$

$$\frac{dQ}{dt} = -k_{quench}QS + (Q_0 - Q)k_r \qquad (S3)$$

The terms in these equations are defined as follows:

Species:	S	- exciton concentration
	Q	- concentration of active quenching sites
	Q_0	- total concentration of quenching sites
Rate constants:	I_0	- exciton generation rate
	τ	- exciton lifetime
	k_q	- exciton quenching
	k_r	- reactivation of quenching sites following a quenching event (inverse trap lifetime).
	k_b	- singlet-singlet annihilation

As in the charge generation models, the exciton lifetime τ is taken to be 260 ps. From the published exciton diffusivity,[6] we estimate $k_{quench} \approx 4\pi R_{quench} D = 4\pi \cdot (1\ \mathrm{nm}) \cdot (0.054\ \mathrm{cm^2s^{-1}}) = 6.8 \times 10^{-8}\mathrm{cm^3s^{-1}}$, where the quenching radius R_{quench} has been taken as 1 nm (the same value used to derive D). The corresponding bimolecular annihilation constant $k_b = 1.35 \times 10^{-7}\ \mathrm{cm^3s^{-1}}$ is a similar magnitude to the charge generation model presented herein.

Intensity-Dependent PL

We begin by setting k_r equal to 0. For a 600 ps pulse, trap lifetimes longer than 1 ns result in essentially identical quenching behaviour. Further, decreasing the trap lifetime requires an increase in the quenching rate to saturate the same concentration of sites. However, we will show that k_q is too large, even when k_r is assumed negligible.

Setting $k_q = 6.8 \times 10^{-8}$ cm^{3}s^{-1} and varying Q_0 in the range 10^{15}-10^{18} cm^{-3} reveals that the rise in PL yield cannot be captured by trap state-filling (Supplementary Fig. 14). In these calculations, we have set $k_b = 1.35 \times 10^{-7}$ cm^{3}s^{-1}.

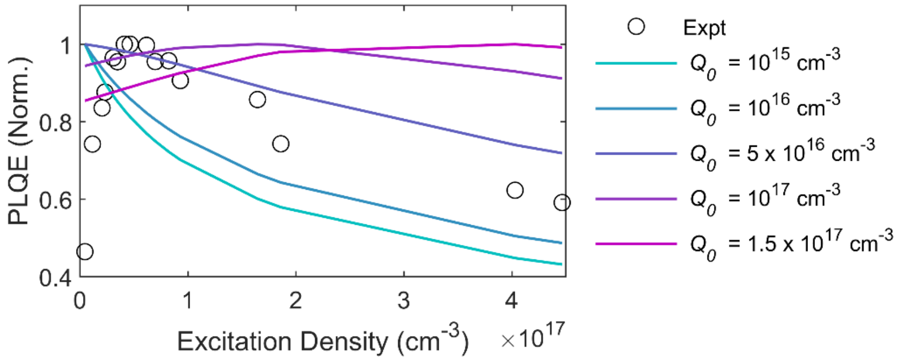

Supplementary Figure 14. Comparison of intensity-dependent photoluminescence to state-filling model, varying trap concentration Q_0. Curves were obtained by numerical integration of the rate equations, with $\tau = 260$ ps, $k_q = 6.8 \times 10^{-8}$ cm^{3}s^{-1}, $k_b = 1.35 \times 10^{-7}$ cm^{3}s^{-1}, and $k_r = 0$ s^{-1}.

By varying both Q_0 and k_q, we can fit the rise in PL intensity, however for the peak position to be correct, a low trap density of $Q_0 = 10^{16}$ cm^{-3} is needed. For this low trap concentration to efficiently harvest excitons at low fluences requires a very large quenching rate $k_q = 6.8 \times 10^{-7}$ cm^{3}s^{-1} (Supplementary Fig. 15) which corresponds to a diffusion coefficient that is an order of magnitude larger than the reported value.[6] The effects of lowering the quenching rate to a factor of 5 larger than expected ($k_q = 3.4 \times 10^{-7}$ cm^3 s^{-1}) and doubling the quencher concentration are also shown.

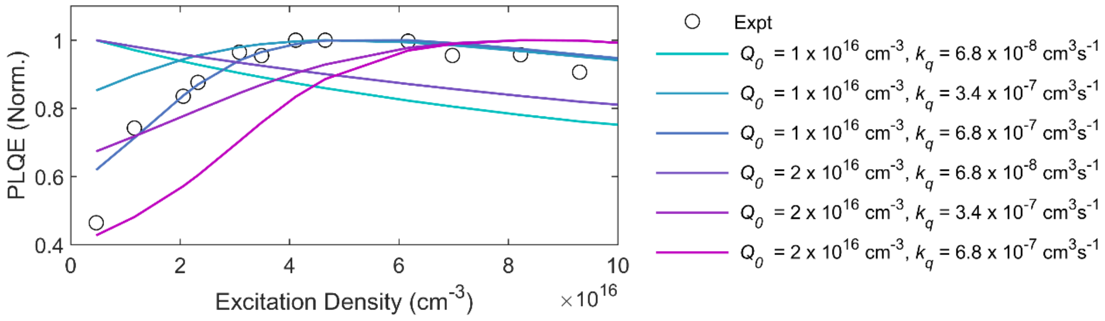

Supplementary Figure 15. Comparison of intensity-dependent photoluminescence to state-filling model, varying trap concentration Q_0 and quenching rate k_q. Curves were obtained using $\tau = 260$ ps, $k_b = 1.35 \times 10^{-7}$ cm^{3}s^{-1}, and $k_r = 0$ s^{-1}.

The application of the trap state-filling model to our PL data can be summarised as follows. To explain the rise in I at low excitation densities (and the resulting peak position), the total trap concentration Q_0 needs to be low enough that increasing intensity in this regime leads to a significant depletion of the concentration of active traps Q. However, fast quenching rates are required for such low trap densities to quench a significant fraction of excitons (which would also be very surprising given the high photovoltaic efficiency of Y6-based devices at solar intensity). These quenching rates conflict with the reported exciton diffusivity for Y6. Shorter trap lifetimes $1/k_r$ require even faster quenching rates to saturate a given trap population.

We now consider the TA data under this model. In Supplementary Fig. 16 we compare the experimentally measured singlet kinetics (which our ultrafast PL measurements confirm as the emissive species) with those predicted by the state-filling model (the non-emissive species is also shown). The modelled singlet kinetics explore 10 equally-spaced values for each parameter, in the ranges required for a PL rise due to trap state-filling (Q_0 = 0.5-2.5 × 10^{16} cm^{-3} and k_q = 3.4-6.8 × 10^{-7} cm^3s^{-1}, k_b = 1.35-4.7 × 10^{-7} cm^3s^{-1}). We note that k_b was varied independently of k_q in these fits, to account for a possible difference in quenching radii for exciton-exciton and exciton-trap quenching (however these two rate constants should still scale together). The modelled kinetics are in quantitative and qualitative disagreement with the exciton kinetics as probed by TA and ultrafast PL.

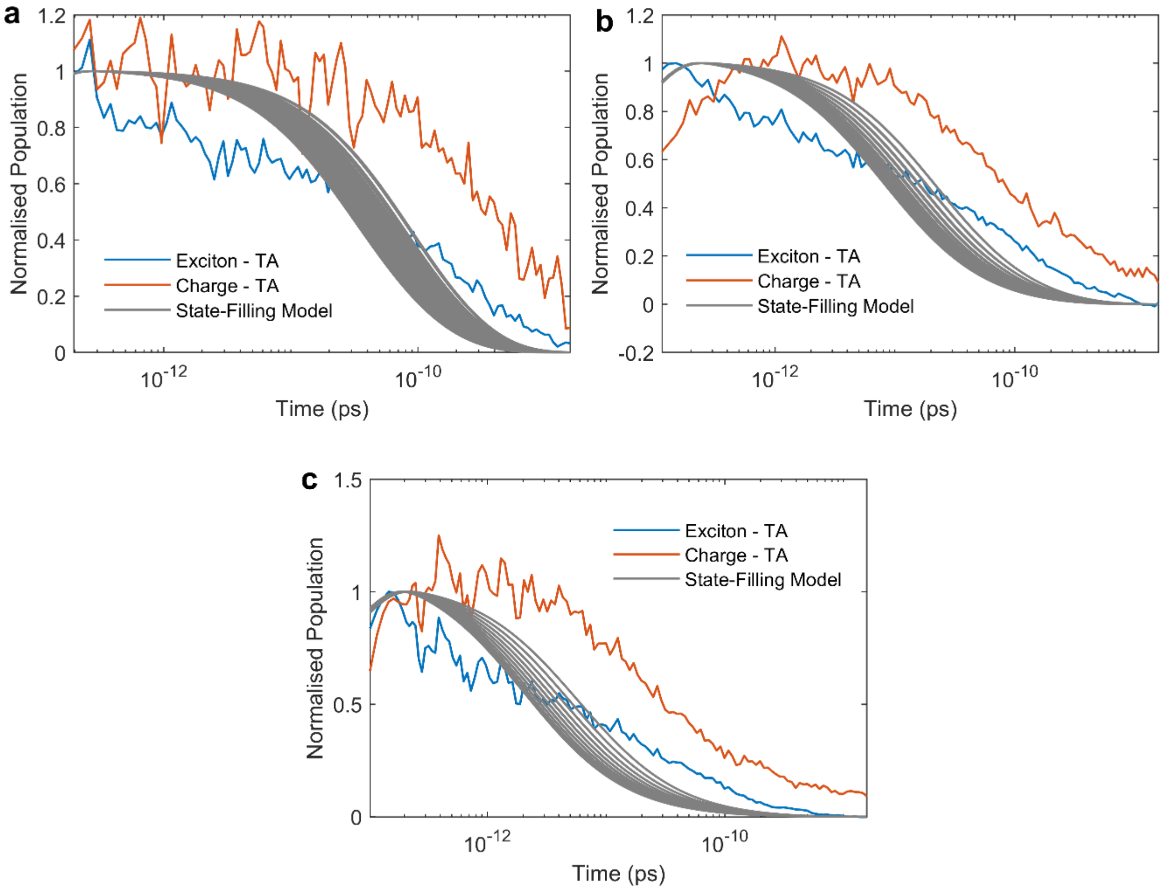

Supplementary Figure 16. Comparison of predicted exciton decay kinetics from trap state-filling model, with transient absorption data at different excitation densities. a) Excitation density = 5 × 10^{16} cm^{-3} . **b)** Excitation density = 2.5 × 10^{17} cm^{-3} . **c)** Excitation density = 1 × 10^{18} cm^{-3}. Curves were obtained with τ = 260 ps, Q_0 = 0.5-2.5 cm^{-3}, k_q = 3.4-6.8 × 10^{-7} cm^3s^{-1}, k_b = 1.35-4.7 × 10^{-7} cm^3s^{-1}, and k_r = 0 s^{-1}.

The disagreement between the state-filling model and the TA dynamics can be summarised as follows: for the PL rise to occur in the observed position, the trap density is required to be ~10^{16} cm^{-3}. This is 5-fold lower than the lowest excitation density used in TA, which means that the exciton dynamics should be largely unaffected by traps. This means that the TA data should provide an accurate measure of the exciton-exciton diffusion coefficient. However, this value is inconsistent with that required to fit the PL data.

These discrepancies rule out excitonic trap filling as the explanation for our measured data. Further reasons for rejecting this hypothesis are:

1. If the trap population is to have such an effect on the exciton radiative efficiency, this effect should appear in the transient exciton kinetics. With decreasing excitation fluence (below the bimolecular recombination threshold), we see a 3-fold decrease in PLQE (Figure 1 a), implying an increase in the non-radiative rate, which necessarily leads to a threefold decrease in the total lifetime. In all of our kinetic data, lifetimes either lengthen with decreasing fluence, or reach a linear regime below which the kinetic is

independent of fluence. Literature values of lifetimes from time-correlated-single-photon counting, [7,8]and Streak camera measurements,[9]which have reached much lower excitation intensities, also fail to show any decrease or non-linearity with lower excitation densities, instead showing consistent lifetimes of ~1.2-1.6 ns.

2. OPV devices with Y6 as the acceptor show very high performance at far below 1 sun incident light intensities.[10] This performance would be expected to drop off as traps become unsaturated at low light levels, but no such effect is seen.

Supplementary Note 3. Comment on triplet yields in neat Y6

The data in Supplementary Fig. 6 shows that triplets are generated in neat Y6, and is consistent with the data of Gillet *et al.* [12], which also shows significant triplet signal after 1000 ps in neat Y6. Also in agreement with Gillet *et al.*, we find no evidence for triplet formation in dispersed Y6:PS blends, indicating that the triplet origin is intermolecular in nature – i.e. from charge recombination. The triplet yield in neat Y6 is much lower than the yield in PM6:Y6 blends, as shown by Gillet *et al.* While this may at first appear surprising, it is also consistent with the key result of Gillet *et al.* which is that triplet exciton formation is suppressed in blends where there is a large difference between the triplet exciton and (interfacial) CT state energies, even though charge formation is highly efficient. The same condition is met in neat Y6: our calculations reveal that the intermolecular CT state is near resonant with the singlet exciton state, and thus much higher in energy than the triplet exciton state. Thus, in line with the arguments presented by Gillet *et al.*, we find that charge recombination efficiently populates the singlet exciton (producing our signature increase in photoluminescence at high excitation density), with less triplet exciton formation than would naively be expected.

Supplementary Note 4. Optical-pump-terahertz-probe spectroscopy

We perform optical-pump-terahertz probe (OPTP) spectroscopy[13,14] on neat films of Y6. Supplementary figure 17a shows the terahertz kinetics of a film excited at approximately 5×10^{18} cm^{-3} initial excitation density, showing similar kinetics to transient absorption kinetics taken at approximately the same fluence. Supplementary Figure 17b shows the conductivity spectrum at 3 ps probe delay of a neat film of Y6, excited with an 800 nm pump beam, at an excitation density of ~1×10^{18} excitations/cm^3. The non-zero real part of the terahertz (THz) spectrum (light blue line) is indicative of a fraction of free polarons, rather than purely bound excitons.[13,15,16] The conductivity spectrum is well described by a fit to a modified Drude-Smith model (DSM) – an established description for charge carriers in pi-conjugated materials.[13,15,16]

$$\tilde{\sigma}(\omega) = \frac{\varepsilon_0 {\omega_P}^2 \tau}{1 - i\omega\tau}\left[1 + \frac{c_1}{1 - i\omega\tau}\right] \qquad (S4)$$

Where ε_0 is the vacuum permittivity, ω_P the plasma frequency, τ the scattering time, and c_1 is the ‘localisation’ parameter. A best fit to our data is obtained with a scattering time of τ = 12 fs, a plasma frequency of ω_P = 20 Thz, and c_1= -0.99. The localisation parameter indicates carriers that are well-localised, consistent with other reports in small molecules and conjugated polymers, and with our computational results presented below. The fitted plasma frequency, ${\omega_P}^2 = \frac{nq^2}{\varepsilon_0 m_e}$, can be used to estimate a very approximate effective mass. Assuming 25% charge yield in line with our models, gives an effective mass of $m^* \sim 1\, m_e$.

This terahertz data is consistent with a portion of free charges present at these high excitation densities, however the high noise level and high excitation densities mean that the data could also be fit with a purely excitonic model as well. Further study on the dynamic terahertz properties of Y6, if able to be performed at sufficiently low signal-to-noise levels, may reveal more useful information.

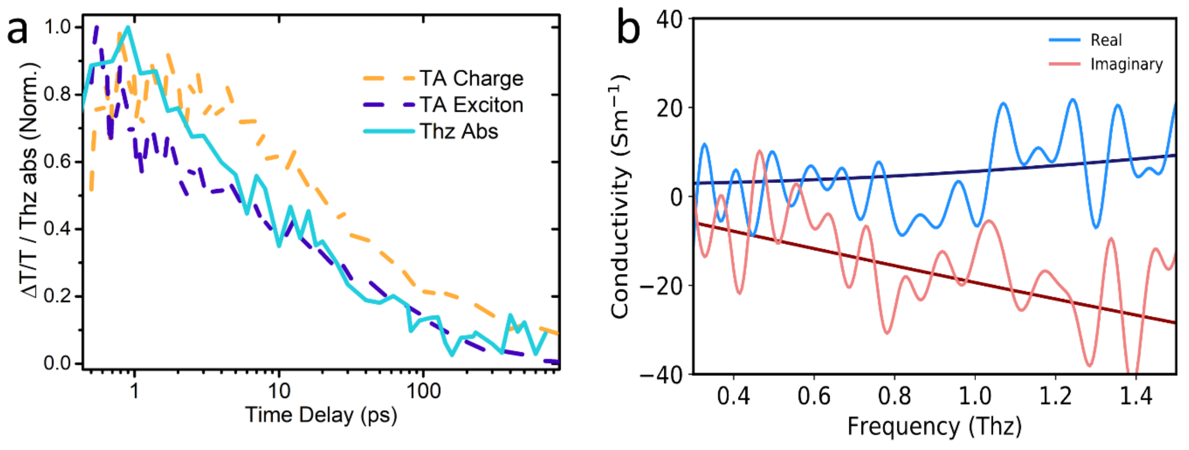

Supplementary Figure 17. Optically-pumped time-resolved terahertz absorption kinetic of neat Y6, compared to transient absorption kinetic decays of excitons and charges. a) The Terahertz measurement is pumped by 800 nm, 150 fs pulses. The roughly matching Thz, and transient absorption kinetics indicate that the two measurements are at roughly the same excitation density, calculated as 5×10^{18} cm^{-3} for the Thz measurement, and 6×10^{18} cm^{-3} for the transient absorption measurement. **b)** Excited-state terahertz complex conductivity spectrum, excited with 150 fs, 800 nm light, giving approximate excitation density 1×10^{18} excitations/cm^3, with a THz probe delay of ~3 ps. The positive real (light blue) line is indicative that there are charge-like species present in addition to purely excitonic photoexcitations. The experimental data is fit to a modified Drude-Smith model (darker blue and darker red curves).

Supplementary Note 5. Kinetic models of exciton and free charge signals in transient absorption and fluence dependent PLQE

Basic Model

In this section we provide a detailed description of the kinetic model used to assign free charge generation in Y6. To minimise the complexity inherent in exciton-mediated charge formation, our model contains only essential processes. Wherever possible, rate constants are estimated based on independent data, rather than being treated as free parameters.

We consider an initial population of delocalised excitons ($S*$) which undergo rapid relaxation to localised excitons (S). Free charge (C) formation from $S*$ competes with relaxation to S, from which charge formation does not occur or has a very slow rate. The separated charges can undergo trap-assisted non-radiative recombination (i.e. Shockley-Read-Hall),[9,17] or recombine bimolecularly to singlet or triplet (T) excitons in a ratio governed by spin statistics. Charge transfer (CT) states are not included as charge recombination is diffusion limited.

$$\frac{dS^*}{dt} = I_0 - (k_{cs} + k_r)S^* - k_b S^*(S + S^*) \qquad (S5)$$

$$\frac{dS}{dt} = k_r S^* - \left(\frac{1}{\tau} + k_b(S + S^*)\right)S + \frac{1}{4}k_{cr}C_e C_h \qquad (S6)$$

$$\frac{dC_{\frac{e}{h}}}{dt} = k_{cs}S^* - k_{tr}C_{\frac{e}{h}} - k_{cr}C_e C_h \qquad (S7)$$

The terms in Equations S5-7 are defined as follows:

Species:	$S*$	- delocalised exciton
	S	- localised exciton
	$C_{e/h}$	- free charges (electrons/holes)
Rate constants:	τ	- exciton lifetime
	k_{cs}	- charge separation
	k_r	- exciton relaxation
	k_{cr}	- bimolecular charge recombination
	k_b	- bimolecular singlet annihilation
	k_{tr}	- pseudo first-order charge decay
	I_0	- exciton generation rate

Although numerous, each of these terms is necessary to properly describe the photo-physics of charge formation and recombination - just as they are for blend systems. Our model depends on four free parameters (k_{cs}, k_{cr}, k_b, k_{tr}), which can be motivated using independently reported data. We now provide detailed descriptions and justifications of these terms.

Fitted and Estimated Rate Constants

***k_r*, *k_{cs}* :** From Figure 3, the charge signature is present from the earliest time delay and prompt exciton decay occurs over the first 1-2 ps, meaning that the rate constants for charge transfer and exciton relaxation must be on the order of 10^{12}-10^{13} s^{-1}, faster than all other processes considered. Comprehensive TA measurements with a wider spectral window in the IR and shorter time resolution[18] have shown that the bulk of charge formation (interpreted as an intra-moiety excited state in Ref 17) occurs within the instrument response, followed by delayed interconversion with a time constant of ~0.2 ps, which we take as the relaxation/localisation rate, $k_r = 5 \times 10^{12}$ s^{-1}. These measurements were based on single wavelength kinetics; however, our spectral decomposition analysis reveals that the exciton population is not completely depleted. This means that fast exciton-to-charge conversion largely occurs prior to relaxation (our model does not include slower CS from relaxed excitons, but we do not exclude this as a possibility). The fitted CS rate is $k_{cs} = 1.8 \times 10^{13}$ s^{-1}.

***k_{cr}* :** The rate constant for bimolecular charge recombination is found to be $k_{cr} = 4.3 \times 10^{-8}$ cm^3s^{-1}, in close agreement with the predicted Langevin rate (1.95×10^{-8} cm^3s^{-1}) based on electron and hole mobilities obtained from thin-film transistor measurements ($\mu_e = 0.004$ $cm^2V^{-1}s^{-1}$, $\mu_h = 0.05$ $cm^2V^{-1}s^{-1}$).[6] We note that macroscopic mobilities derived from device measurements include grain boundary and other effects, which means these values serve as lower bounds to the local charge mobilities, which are expected to be larger.

***k_{tr}* :** The rate constant for pseudo first-order non-radiative charge decay is fit to $k_{tr}=2\times10^8$ s^{-1}, which is consistent with a trap density of ~10^{16} cm^{-3} using our value of k_{cr} given above. This is consistent with literature estimates of trap density.[19]

***k_b* :** The rate constant for exciton-exciton annihilation k_b takes an optimal value of 3×10^{-7} cm^3s^{-1}. This is comparable to the previously reported value of 1.35×10^{-7} cm^3s^{-1}, with the difference being that the literature value assumes an excitonic picture, and therefore takes an intermediate value between our bimolecular exciton and charge annihilation constants k_b and k_{cr}.

***τ* :** The exciton lifetime τ is estimated as 260 ps, based on the excited state lifetime of Y6 in solid solution (polystyrene), which also is comparable to that observed in dichloromethane.[18]

For TA measurements, the excitation profile I_0 is a gaussian pulse normalised to the experimental excitation density. The temporal duration (FWHM) was 200 fs for TA, and 600 ps for intensity-dependent PL.

The fit of this basic model to the data is shown in the main text Figure 3. With only 4 free parameters, our model globally fits our set of 6 different transient absorption kinetics, and fluence dependent PL yield curve well.

We use the model above to put conservative bounds on the minimum/maximum steady-state free charge fraction, at one sun (AM1.5) light intensity, and on the probability of an excited exciton dissociating to a charge at some point in its lifetime. For a wide range of the above recombination constants, we calculate the theoretical PLQE. If the PLQE at an excitation density of 10^{17} cm^{-3} is 10% greater than the PLQE at 10^{13} - 10^{15} cm^{-3}, we calculate the error

associated with the global fit of the kinetic model to the fluence-dependent transient absorption kinetics of singlets and free charges. If the calculated PLQE shows no rise with increasing excitation density, it is not consistent with the rise shown in our experimental PLQE data, and therefore is not included for error analysis. Supplementary Figure 18 shows the results of this analysis. The exciton to charge dissociation probability fits into a narrow range of ~75-90%, and the steady-state free charge fraction at 1 Sun shows a broader range (~65-90%). In line with the steep slope from the Saha equation seen in main text Figure 3 e.

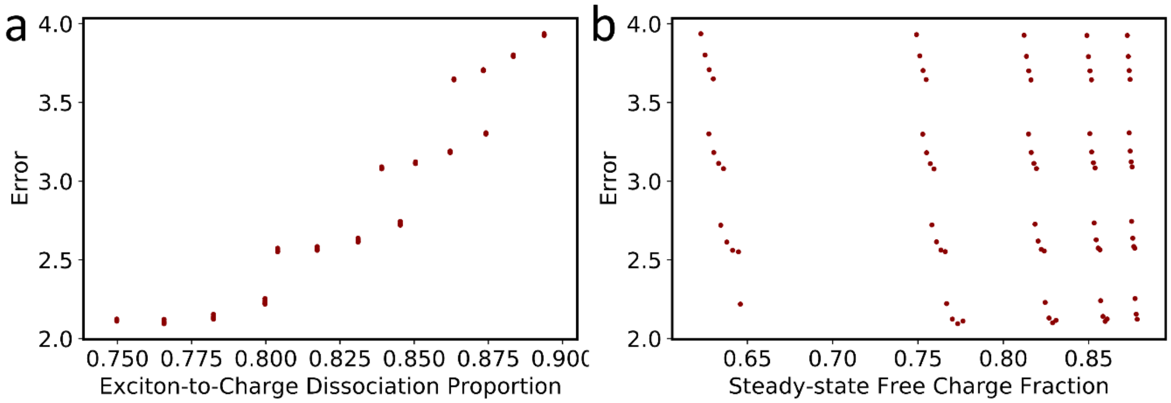

Supplementary Figure 18. Exciton dissociation, and steady state charge fractions with error of fit to TA and PL data for the Basic Model. a) The proportion of excitons that dissociate to charges at some point during their lifetime, plotted against the error of the modelled TA from the experimental TA – if the model also produces at least a 10% rise in PL with increasing fluence. **b)** The steady-state free charge fraction plotted against the TA fit error. Kinetics were normalised before calculating the error. The error is calculated as described in the above section, specifying the criteria as needing a 10% rise in PLQE.

Model Including Triplet-Triplet Annihilation

Our basic model treats recombination of triplet CT excitons via back transfer as a loss pathway, This approach is justified when describing the TA dynamics, which cover a timescale of 0-1.6 ns and are dominated by charge formation recombination and monomolecular exciton decay. However, repopulation of the emissive singlet state via triplet-triplet annihilation (TTA) could potentially affect the intensity-dependence of the PL.

Explicit consideration of triplets requires the introduction of rate constants for TTA (k_{tta}), and monomolecular triplet decay (k_{td}). The corresponding rate equations are:

$$\frac{dS^*}{dt} = I_0 - (k_{cs} + k_r)S^* - k_b S^*(S + S^*) \qquad (S8)$$

$$\frac{dS}{dt} = k_r S^* - \left(\frac{1}{\tau} + k_b(S + S^*)\right)S + \frac{1}{4}k_{cr}C_e C_h + \frac{1}{2}k_{tta}T^2 \qquad (S9)$$

$$\frac{dC_{\frac{e}{h}}}{dt} = k_{cs}S^* - k_{tr}C_{\frac{e}{h}} - k_{cr}C_e C_h \qquad (S10)$$

$$\frac{dT}{dt} = \frac{3}{4}k_{cr}C_e C_h - k_{tta}T^2 - k_{td}T \qquad (S11)$$

It is the balance between k_{tta} and k_{td} that determines the possible PL rise, while their absolute values are constrained by the exciton decay dynamics. To ensure comparison with the basic model, we constrain the triplet and singlet exciton annihilation terms such that (k_b > k_{tta}), keeping all other rate constants as in the previous section. We then fit k_b = 6.5 × 10^{-7} cm^3s^{-1} , k_b = 0.6 × 10^{-7} $cm^3s^{-1}$and k_{td} = 1 × 10^8 s^{-1}. As can be seen in Supplementary Figure 19, this improves the quality of the fit to the PL data.

We emphasise that while the inclusion of TTA improves our data fitting, it cannot provide a satisfactory explanation of our data without free charge generation. This is because TTA can only affect the PL data if the number of triplets formed is very large. Triplet formation therefore needs to occur rapidly, since it is limited by the singlet lifetime (~260 ps). This timescale is likely to be out of reach for intersystem crossing (and could not give a bimolecular dependence of the PL intensity on excitation density), leaving singlet fission and charge recombination as the two possible mechanisms. However, the TA spectrum of Y6 is dominated by two species – the singlet (which follows the TRPL decay), and a second species. For TTA to replace charge generation as an explanation of our data, the second species that we have assigned as charges would need to be reinterpreted as a triplet signature. However, this interpretation is ruled out by several considerations. Specifically, the non-singlet spectral signature in neat Y6:

- has been shown to precede hole injection in donor/Y6 blends[18]

- closely resembles the electron signature in donor/Y6 blends[18] (as also demonstrated in the main text)
- is dominated by a negative near band-edge feature, which suggests electro-absorption.
- is distinct from the triplet signature observed in 26ensitized Y6 films[12]

Transient absorption measurements of PtOEP:Y6 show a large number of triplets,[20] implying a triplet energy level of above 1.1 eV, and hence singlet fission cannot occur from thermalised Y6 singlet excitons. Additionally, we know that the peak internal PLQE of a Y6 film – estimated in this paper as ~6% - is close to the estimated PLQE of dilute Y6 in a solid solution of polystyrene (~7%) – although these values have significant error associated with them.

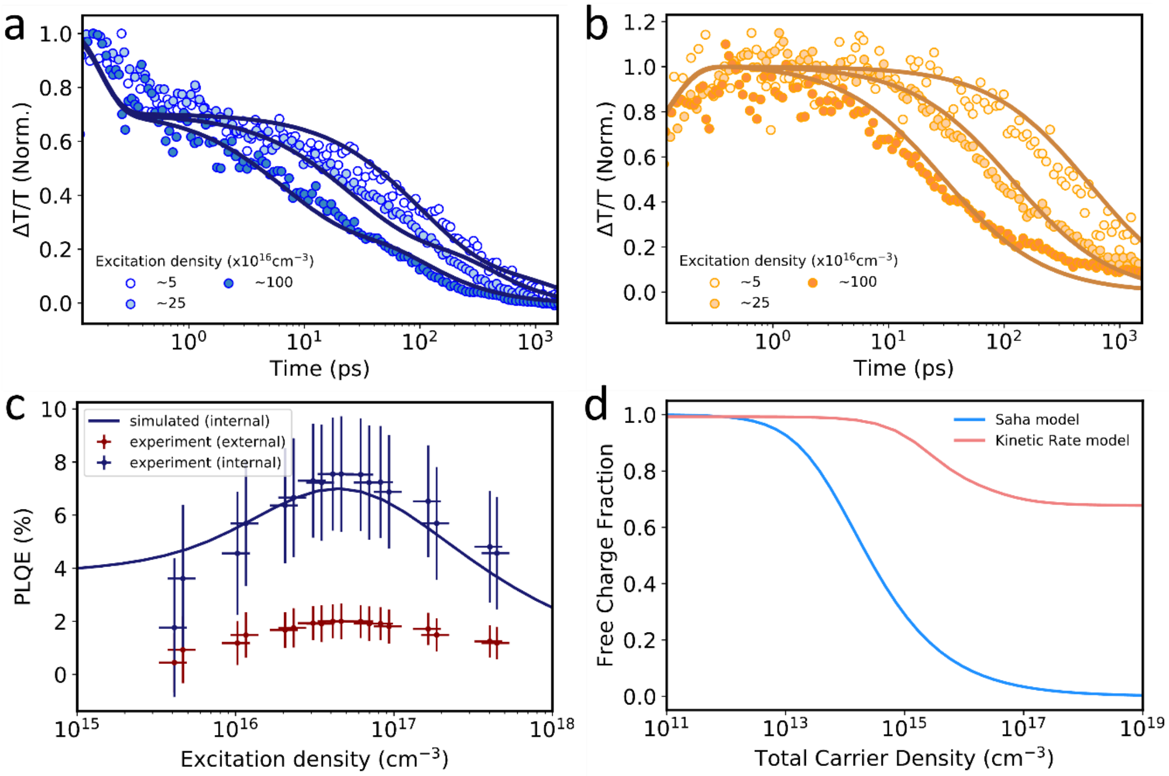

Supplementary Figure 19. Kinetic model, including triplet-triplet annihilation, of charge generation in Y6 with transient absorption, PLQE and predicted steady-state free charge fractions. a) Normalised transient absorption kinetics of excitons (blue circles) and **b)** charge states (orange circles) at different excitation densities, fitted with a global fit to the kinetic model described above which includes TTA (solid blue and orange lines) **c)** Internal (blue crosses) and external (red crosses) PLQE values of Y6 as a function of excitation density, with estimated error. Simulated internal PLQE (blue line) is calculated from fits to the transient absorption and intensity dependent PLQE. **d)** Steady-state free charge fraction as a function of total excitation density calculated from the rate constants gathered from the kinetic model (red line), and from an estimate from the Saha equation (blue line). The Saha equation is calculated based on an excitonic binding energy of 270 meV, corresponding to an exciton effective mass of ~0.5 me.

Supplementary Figure 20 shows error analysis to give an error bound of the exciton dissociation probability and free charge fraction at 1 Sun intensity. The exciton to charge dissociation probability fits into a range of ~60-90%, and the steady-state free charge fraction at 1 Sun shows a charge fraction above 95%.

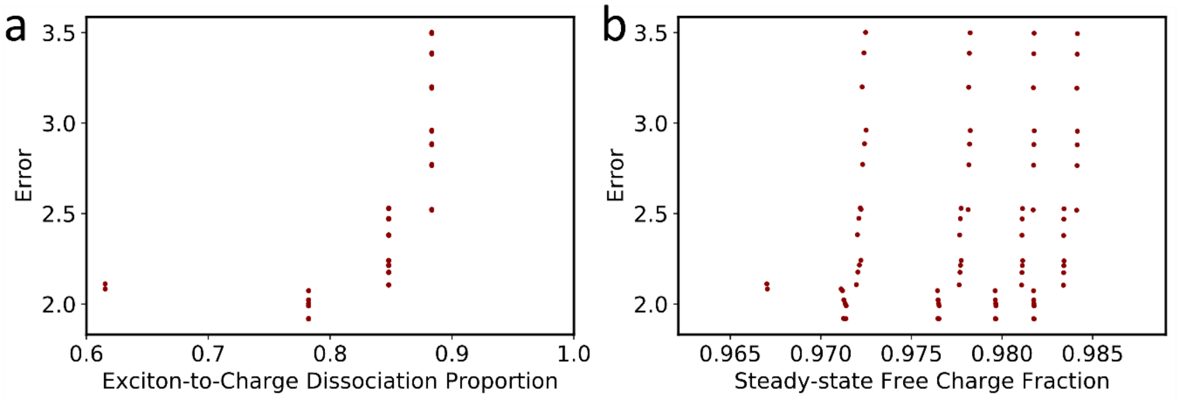

Supplementary Figure 20. Exciton dissociation, and steady state charge fractions with error of fit to TA and PL data for the Basic Model with TTA included. A) The proportion of excitons that dissociate to charges at some point during their lifetime, plotted against the error of the modelled TA from the experimental TA – if the model also produces at least a 10% rise in PL with increasing fluence. **B)** The steady-state free charge fraction plotted against the TA fit error. Kinetics were normalised before calculating the error. The error is calculated as described in the basic kinetic model section, specifying the criteria as needing a 10% rise in PLQE.

Model Including Finite Charge Trap Population

Another potential contribution to the PL rise is a saturable charge trap population (excitonic traps are considered in an above section). At low excitation densities a fraction of photogenerated charges are quenched by trap sites rather than contributing to the PL yield. At high excitation densities the trap sites are saturated, leading to an increase in the PL yield. We include this in our model by adding a trap population (Q), which can be depleted by encounters with charges. Similar results are obtained for including both hole and electron traps, however we show here only hole trap contributions. This keeps parameters to a minimum, and is justified considering that Y6 is intrinsically *n*-doped, and literature shows good evidence for hole trap saturation in Y6 mobility measurements.[21]

$$\frac{dS^*}{dt} = I_0 - (k_{cs} + k_r)S^* - k_b S^*(S + S^*) \qquad (S12)$$

$$\frac{dS}{dt} = k_r S^* - \left(\frac{1}{\tau} + k_b(S + S^*)\right)S + \frac{1}{4}k_{cr}C_e C_h \qquad (S13)$$

$$\frac{dC_e}{dt} = k_{cs}S^* - k_{cr}C_e C_h \qquad (S14)$$

$$\frac{dC_h}{dt} = k_{cs}S^* - k_{cr}C_e C_h - k_{cr}C_h Q \qquad (S15)$$

$$\frac{dQ}{dt} = -k_{cr}C_h Q \qquad (S16)$$

In this treatment, the pseudo first order charge decay constant k_{tr} from the basic model has been replaced with the bimolecular charge recombination constant k_{cr} which governs the encounter rate of free charges with one another, and with charge traps. We retain k_{cs} from the basic model, and fit k_{cr}, k_b, and Q_0 – the initial trap population. It should be noted that these equations identify the traps as negative charges (to ensure charge conservation) that are present due to intrinsic/unintentional doping, which are taken to be mobile under continuous excitation conditions, and in view of the low level of energetic disorder.

The fitted parameters are k_{cr} = 4 ×10^{-8} cm^3s^{-1}, k_b = 4.7 ×10^{-7} cm^3s^{-1}, and Q_0 = 2 × 10^{16} cm^{-3}, corresponding fits are shown in Supplementary Figure 21. k_{cr} and k_b are in the expected range. The trap population is also in a physically acceptable range, given the value of 8 × 10^{16} cm^{-3} reported by Zhang *et al.*,[19] and the dependence of trap concentration on film preparation conditions. We note that with these fitted parameters, if the trap concentration is set to zero, no

rise in PL can be obtained. This again highlights the sensitivity of our models to the experimental data.

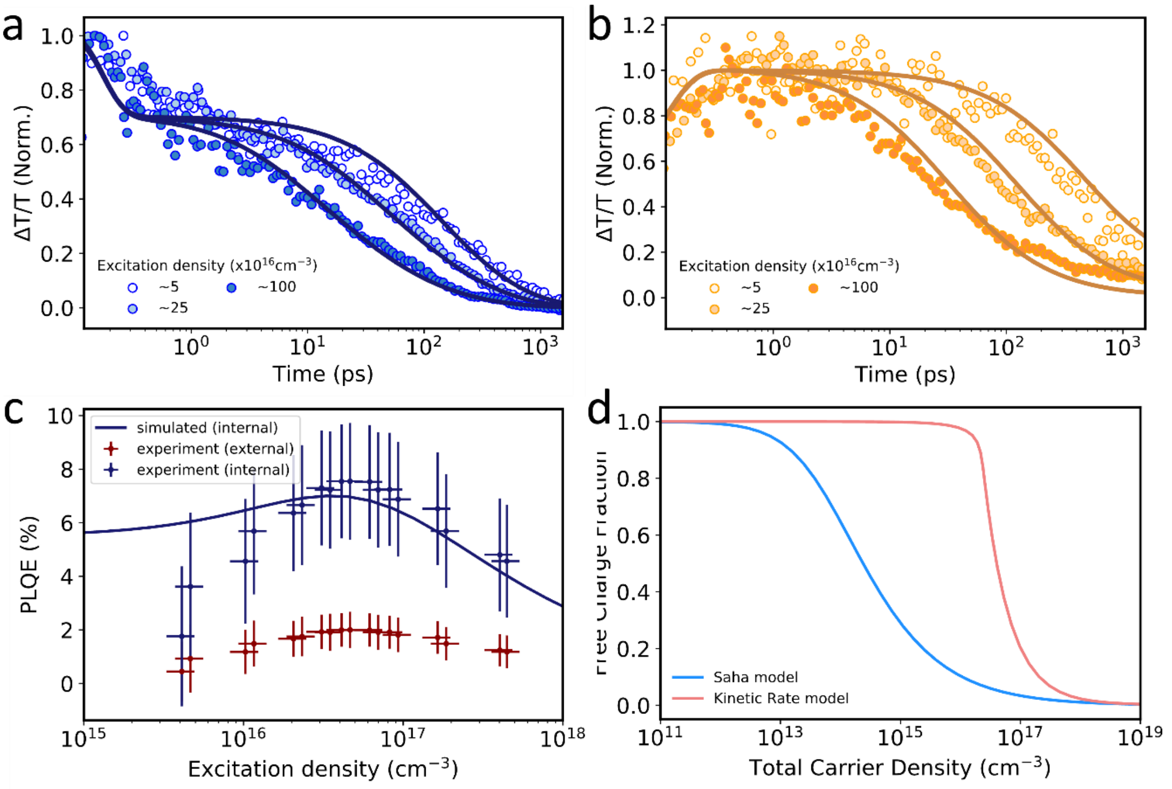

Supplementary Figure 21. Kinetic model, including hole trap saturation, of charge generation in Y6 with transient absorption, PLQE and predicted steady-state free charge fractions. a) Normalised transient absorption kinetics of excitons (blue circles) and **b)** charge states (orange circles) at different excitation densities, fitted with a global fit to the basic kinetic model described above (solid blue and orange lines) **c)** Internal (blue crosses) and external (red crosses) PLQE values of Y6 as a function of excitation density, with estimated error. Simulated internal PLQE (blue line) is calculated from fits to the transient absorption and intensity dependent PLQE. d) Steady-state free charge fraction as a function of total excitation density calculated from the rate constants gathered from the kinetic model (red line), and from an estimate from the Saha equation (blue line). The Saha equation is calculated based on an excitonic binding energy of 270 meV, corresponding to an exciton effective mass of ~0.5 m_e.

Supplementary Figure 22 shows error analysis of the charge trap model to give an error bound of the exciton dissociation probability and free charge fraction at 1 Sun intensity. The exciton to charge dissociation probability fits into a range of ~75-90%, and the steady-state free charge fraction at 1 Sun shows a charge fraction above 88%.

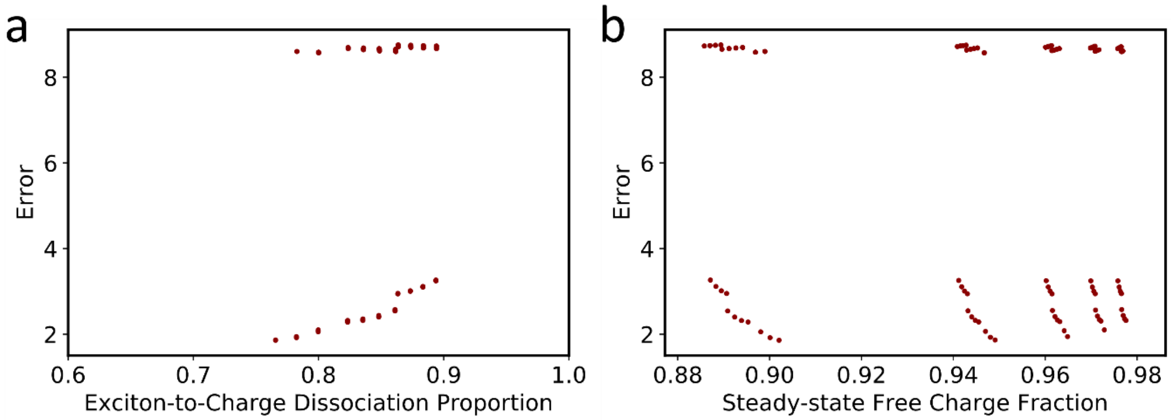

Supplementary Figure 22. Exciton dissociation, and steady state charge fractions with error of fit to TA and PL data for the Basic Model with hole traps included. a) The proportion of excitons that dissociate to charges at some point during their lifetime, plotted against the error of the modelled TA from the experimental TA – if the model also produces at least a 10% rise in PL with increasing fluence. **b)** The steady-state free charge fraction plotted against the TA fit error. Kinetics were normalised before calculating the error. The error is calculated as described in the basic kinetic model section, specifying the criteria as needing a 10% rise in PLQE.

Consideration of Explicit Singlet-CT State Equilibrium

In addition to the above models, we have considered a model that explicitly models CT states, and that considers the 'charge' species in the TA to be initially entirely composed of CT states. We can model the early time evolution of the two TA species, over the first ~10 ps, as arising from a rapidly established equilibrium of CT states with singlet excitons, and this model can match the TA data over the time period from 0 - 200 ps delay. Under this model, charges can form thermally from the CT states on a slower time scale. Best fits of this model to the data give exciton dissociation probabilities of 25-40%, and steady-state charge fractions of 70-85%. However, models which form enough charges to induce a rise in PL with fluence, are unable to adequately globally fit the TA kinetics at a time beyond ~200 ps. As well, this model neglects charge recombination to triplet formation. When this is included – as either a loss pathway or allowing TTA – the model deviates even further from experimental data.

Supplementary Note 6. Photon Reabsorption in thin films

Photon reabsorption effects in photoluminescence quantum efficiency (PLQE) measurements were quantified as per Richter *et al.*[17] We estimated the escape probability with the same method[17], based on the optical ellipsometric constants measured by Kerremans *et al.*[22], giving a conservative estimate of escape cone loss of $\eta_{esc} = 25\%$. The externally measured PLQE, including effects of reabsorption and photon recycling, is given by,

$$\eta_{ext} = \frac{\eta \cdot \eta_{esc}}{1 - \eta + \eta \cdot \eta_{esc}}$$

Where η is the internal PLQE. With no photon recycling, $\eta_{ext} = \eta \cdot \eta_{esc}$

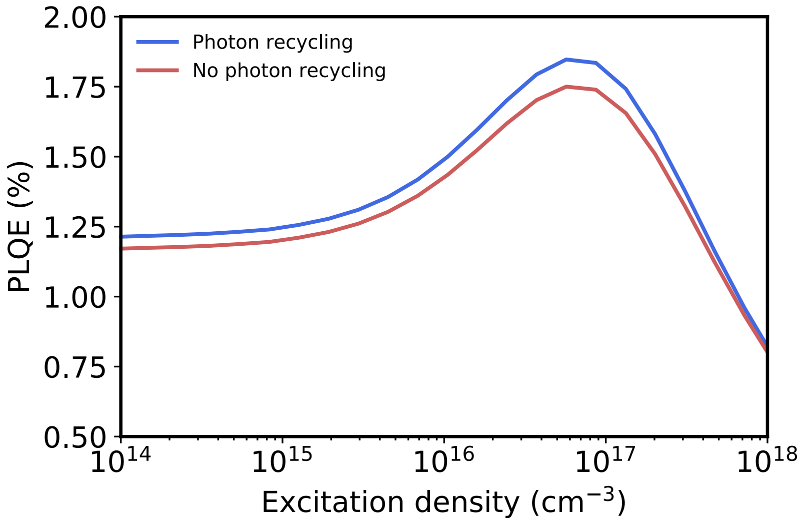

Supplementary Figure 23. Simulated PLQE of Y6 with and without the effect of photon recycling. The simulated, fluence dependent PLQE based on the model shown in Figure 3 of the main text, illustrating the small, but non-zero enhacement in externally measured PLQE expected due to photon-recycling in thin films of Y6.

Supplementary References

1. Zhang, G. *et al.* Single-Junction Organic Solar Cell with over 15% Efficiency Using Fused-Ring Acceptor with Electron-Deficient Core. *Joule* 3, 1–12 (2019).

2. Zhang, M., Guo, X., Ma, W., Ade, H. & Hou, J. A Large-Bandgap Conjugated Polymer for Versatile Photovoltaic Applications with High Performance. *Adv. Mater.* **27**, 4655-4660 (2015).

3. Lin, Y. *et al.* A Facile Planar Fused-Ring Electron Acceptor for As-Cast Polymer Solar Cells with 8.71% Efficiency. *J. Am. Chem. Soc.* **138**, 2973–2976 (2016).

4. Liao, S. H., Jhuo, H. J., Cheng, Y. S. & Chen, S. A. Fullerene derivative-doped zinc oxide nanofilm as the cathode of inverted polymer solar cells with low-bandgap polymer (PTB7-Th) for high performance. *Adv. Mater.* **25**, 4766-4771 (2013).

5. De Mello, J. C., Wittmann, H. F. & Friend, R. H. An improved experimental determination of external photoluminescence quantum efficiency. *Adv. Mater.* **9**, 230–232 (1997).

6. Firdaus, Y. *et al.* Long-range exciton diffusion in molecular non-fullerene acceptors. *Nat. Commun.* 11, 5220 (2020).

7. Zhang, G. *et al.* Delocalization of exciton and electron wavefunction in non-fullerene acceptor molecules enables efficient organic solar cells. *Nat. Commun.* 11, 1–10 (2020).

8. Classen, A. *et al.* The role of exciton lifetime for charge generation in organic solar cells at negligible energy-level offsets. *Nat. Energy* **5**, 711–719 (2020).

9. Zou, X. *et al.* An Insight into the Excitation States of Small Molecular Semiconductor Y6. *Molecules* **25**, 4118 (2020).

10. Ma, L. K. *et al.* High-Efficiency Indoor Organic Photovoltaics with a Band-Aligned Interlayer. *Joule* 4, 1486–1500 (2020).

11. Wang, R. *et al.* A universal approach for optimizing charge extraction in electron transporting layer-free organic solar cells: Via Lewis base doping. *J. Mater. Chem. A* **7**, 25808-25817 (2019).

12. Gillett, A. J. *et al.* The role of charge recombination to spin-triplet excitons in non-fullerene acceptor organic solar cells *Nature*, 597, 666–671 (2020).

13. Krauspe, P. *et al.* Terahertz short-range mobilities in neat and intermixed regions of polymer:fullerene blends with controlled phase morphology. *J. Mater. Chem. A* **6**, 22301–22309 (2018).

14. Hendry, E., Schins, J. M., Candeias, L. P., Siebbeles, L. D. A. & Bonn, M. Efficiency of exciton and charge carrier photogeneration in a semiconducting polymer. *Phys. Rev. Lett.* 92, 196601 (2004).

15. Cooke, D. G., Krebs, F. C. & Jepsen, P. U. Multi-THz spectroscopy of mobile charge carriers in P3HT:PCBM on a sub-100 fs time scale. *Phys. Chem. Interfaces Nanomater. XII* 88111H (2013).

16. Sun, Y. *et al.* Electronic processes investigation from ultrafast terahertz in photovoltaic

DPPDTT-PCBM films. *Sol. Energy Mater. Sol. Cells* 215, 110684 (2020).

17. Richter, J. M. *et al.* Enhancing photoluminescence yields in lead halide perovskites by photon recycling and light out-coupling. *Nat. Commun.* 7, 13941 (2016).

18. Wang, R. *et al.* Charge Separation from an Intra-Moiety Intermediate State in the High-Performance PM6:Y6 Organic Photovoltaic Blend. *J. Am. Chem. Soc.* 142, 12751–12759 (2020).

19. Zhang, Y. *et al.* An Electron Acceptor Analogue for Lowering Trap Density in Organic Solar Cells. *Adv. Mater.* 33, 2008134 ~~1–8~~ (2021).

20. Natsuda, S.-I. *et al.* Singlet and triplet excited-state dynamics of a nonfullerene electron acceptor Y6 *J. Phys. Chem. C* 125, 20806–20813 (2021).

21. Yao, N. *et al.* Efficient Charge Transport Enables High Efficiency in Dilute Donor Organic Solar Cells. *J. Phys. Chem. Lett.* 12, 5039–5044 (2021).

22. Kerremans, R. *et al.* The Optical Constants of Solution-Processed Semiconductors—New Challenges with Perovskites and Non-Fullerene Acceptors. *Adv. Opt. Mater.* 8, 2000319 (2020).

23. Cui, Y. *et al.* A Novel pH Neutral Self-Doped Polymer for Anode Interfacial Layer in Efficient Polymer Solar Cells. *Macromolecules* 49, 8126–8133 (2016).

24. Hsu, C. P., You, Z. Q. & Chen, H. C. Characterization of the short-range couplings in excitation energy transfer. *J. Phys. Chem. C* 112, 1204–1212 (2008).

25. Löwdin, P. O. On the non-orthogonality problem connected with the use of atomic wave functions in the theory of molecules and crystals. *J. Chem. Phys.* 18, 365-375 (1950).

26. Karuthedath, S. *et al.* Intrinsic efficiency limits in low-bandgap non-fullerene acceptor organic solar cells. *Nat. Mater.* 20, 378–384 (2021).
